# Unintended consequences: Assessing thermo-mechanical changes in vinyl nitrile foam due to micro-computed X-ray tomographic imaging

**DOI:** 10.1016/j.matdes.2023.112381

**Published:** 2023-11

**Authors:** Alexander K. Landauer, Zois Tsinas, Orion L. Kafka, Newell H. Moser, Jack L. Glover, Aaron M. Forster

**Affiliations:** a Material Measurement Laboratory, National Institute of Standards and Technology, 100 Bureau Drive, Gaithersburg, 20899, MD, USA; b Material Measurement Laboratory, National Institute of Standards and Technology, 325 Broadway, Boulder, 80305, CO, USA; c Theiss Research, La Jolla, 92037, CA, USA; d Physical Measurement Laboratory, National Institute of Standards and Technology, 100 Bureau Drive, Gaithersburg, 20899, MD, USA

**Keywords:** X-ray modification, Radiation damage, Polymer foam, Impact protection, X-ray micro-computed tomography

## Abstract

Micro-computed X-ray tomography (μCT) is a volumetric imaging tool used to quantify the internal structure of materials. μCT imaging with mechanical testing (*in situ* μCT) helps visualize strain-induced structural changes and develop structure-property relationships. However, the effects on thermophysical properties of radiation exposure during *in situ* μCT imaging are seldom addressed, despite potential radiation sensitivity in elastomers. This work quantifies the radiation dosage effect on thermo-, chemical-, and mechanical-properties for a vinyl nitrile-based foam. Material properties were measured after (0, 1, 2, and 3) days at (8.1 ± 0.9) kGy/d. Morphological characteristics were investigated via scanning electron microscopy. Thermal transitions were assessed using differential scanning calorimetry. Viscoelasticity was measured with dynamic mechanical analysis over a range from −30 °C to 60 °C. Higher dose lead to stiffening and increased dissipation. Chemical structure was assessed with Fourier transform infrared spectroscopy and energy-dispersive X-ray spectroscopy. Soxhlet extraction was used to measure gel content. In summary, substantial changes occur in thermophysical properties, which may confound structure-property measurements. However, this also provides a modification pathway. Quantitation and calibration of the properties changes informed a finite element user material for material designers to explore tunablity and design optimization for impact protection engineers.

## Graphical Abstract

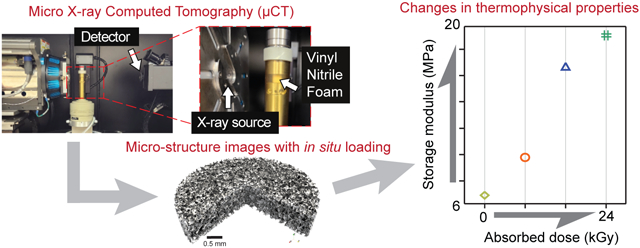

## 1. Introduction

**Fig. 1.**
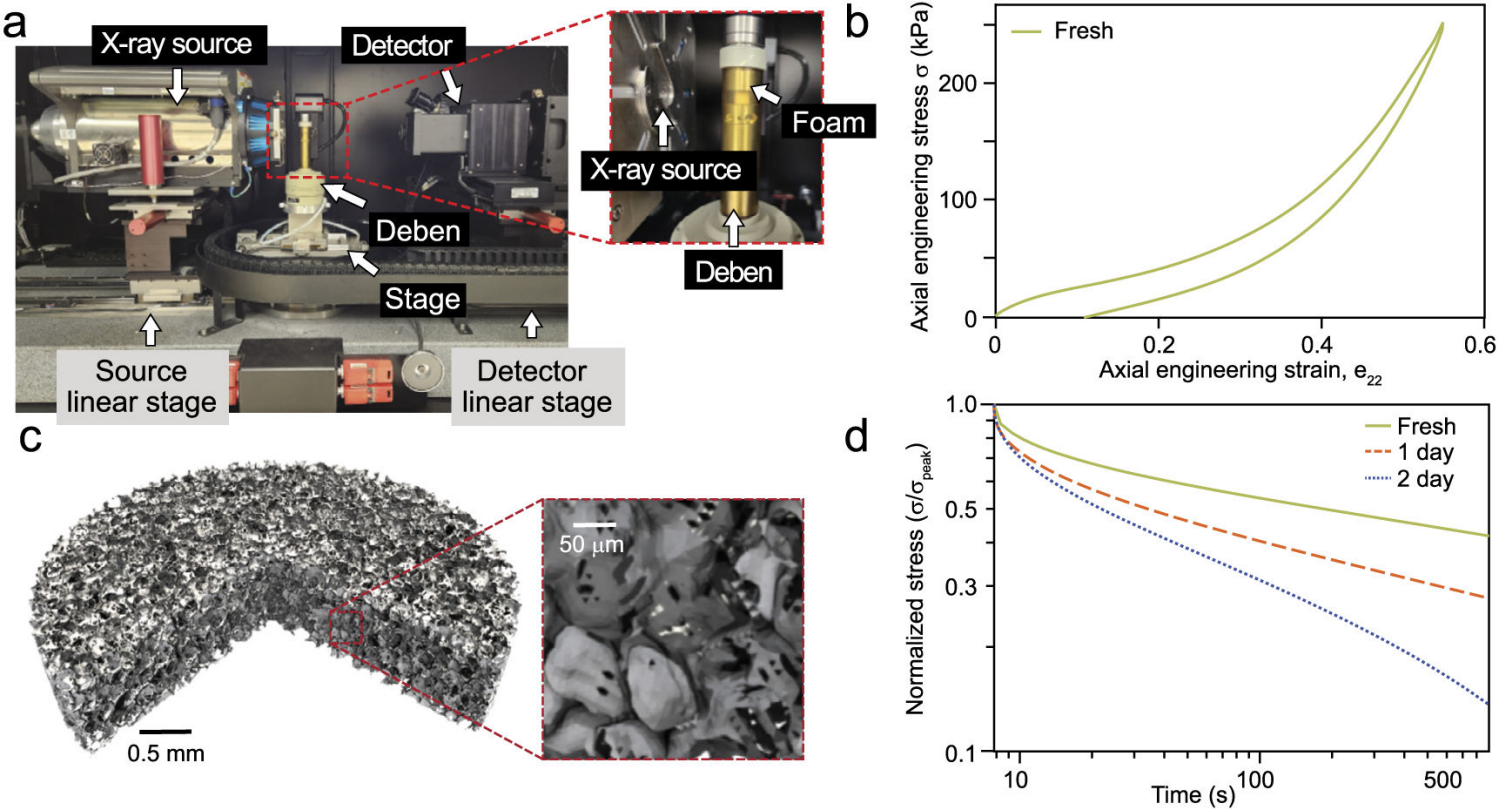
Instrument configuration and baseline material structure and mechanical response. (a) Layout photo of the micro-computed X-ray tomography instrument, labeling the source, specimen, detector configuration. For *in-situ* loading, a cylinder-shaped load frame is introduced in the beam path at the specimen location on the stage. (b) Typical quasi-static strain rate (10^−4^ s^−1^) load-unload stress-strain response of the fresh material with strain uncertainty ± 0.25 % and stress uncertainty ± 5.3 kPa. (c) Rendered virtual core sample of the foam showing the closed-cell microstructure from a typical single scan. (a), (b), and (c) are adapted from data or figures originally in [35], used with permission. (d) Stress relaxation example data from the dynamic mechanical analysis instrument, showing a marked difference between the stress response of fresh and irradiated foams. Stress uncertainty is approximately ± 0.2 kPa.

Micro-computed X-ray tomography (μCT) is a technique for visualizing and measuring the internal structure of optically opaque materials. Lab-scale μCT instruments are sophisticated tools with sub-micrometer-scale voxel size and integrated *in-situ* instrumentation (i.e., devices designed to operate inside the imaging chamber during image) such as environmental stages or load frames [1]. A labeled image of the lab-scale machine used in this study is shown in Fig. 1a. As a quantitative imaging modality, μCT has been widely employed to examine the microstructure of heterogeneous polymer materials across a range of applications, to measure quantities of interest for structure-properties material modeling, and to inform 3D finite element models [2]. Example quantities of interest for foams and polymers include feature sizes (voids, particles, ligaments) [3,4], relative density and porosity [4-7], or anisotropy and auxaticity [8,2]. For polymer foam applications, *in-situ* load frames enable microstructural imaging, stress, and strain measurements, which enable a volumetric assessment of the onset of instabilities, measure materials properties such as the tangent Poisson’s ratio, and apply techniques such as digital volume correlation (DVC) [9,10], typically over the course of several scans [11,12,8,13-15]. For complex or heterogeneous material applications, such as foams used for impact protection [16-18], micro-structural responses to strain are important to modeling the material performance [19,20,11].

Unintended effects from ionizing radiation on the polymer matrix may occur during μCT. The primary interaction mechanism of X-ray electromagnetic radiation with polymers is through secondary electrons from Compton scattering, which initiate ionization events and other chemical reactions [21]. Irradiation of polymeric materials can excite and ionize atoms resulting in the formation of free radicals within the polymer matrix [22]. Radicals can promote the decomposition and/or crosslinking of the polymers, as well as the formation of new functional groups, depending on the surrounding environment [23,24]. In the case of vinyl nitrile foam materials (typically polyvinyl chloride – nitrile butadiene blends), the primary radicals expected to form are carbon centered radicals via the cleavage of an ─H or ─Cl bond along the main chain or the scission of a carbon─carbon bond from the backbone of the polymer, depending on the irradiation parameters (total dose, dose rate, etc.) [25]. When oxygen is present, carbon centered radicals can react with the available oxygen and form alkoxy radicals that are highly reactive and initiate a series of secondary reactions that further decrease the molar mass of the polymer [21,26,27]. Therefore, when techniques that require the use of X-rays to evaluate the morphology and structure of polymers are applied, it is important to study material compatibility to ensure that no significant changes occur in its chemical structure and/or properties during measurement. With the increasingly widespread use of μCT for *in-situ* studies of polymer foams, particularly studies with multiple scans of the same specimen, altering the nature of the material is of increasing concern, as the polymer response to the applied stress (e.g., stress relaxation, buckling, and/or localization behaviors) may change over the course of the measurement. For example, see the difference in stress relaxation response shown in Fig. 1b.

While the effects of radiation on polymers, and more specifically nitrile-butadiene rubber (NBR), poly(vinyl) chloride, and NBR-polymer blends has been extensively studied, this is often for applications that involve much higher radiation doses, for example radiation processing, dosimetry, and nuclear applications [22,28-30]. Possible damage to biological tissue due to μCT radiation exposure has long been recognized [31], and hard tissue damage was characterized by Barth and coworkers [32] in the context of structure-properties investigations. However, quantification for soft tissues (e.g., biopolymers such as collagen) and DVC applications remains limited [33]. The primary mechanisms of polymer modification due to ionizing radiation in biomaterials are crosslinking and chain scission [29,34,22]. These mechanisms are also relevant to non-biologicals, such as polymer blends [27].

Observations during *in-situ* μCT compression testing of vinyl nitrile foam specimens for structure-property analysis and DVC [35] showed that the matrix material underwent qualitative irreversible changes in color, stiffness, and viscoelasticity due to radiation exposure. The X-ray dose (ca. 9kGy for a compression sequence) was relatively low compared to the typical dosage experienced for X-ray processing of polymers [22,36,37] or exposure studies in nuclear applications [28], however, since the base NBR and PVC polymers are known to be radiation sensitive [38] the authors suspected a radiation-based modification occurred in the foam matrix polymer. Although the effects of radiation on thermomechanical properties of other foamed polymers have been studied (e.g., [39,24]), and vinyl nitrile and poly(vinyl) chloride solid polymer structures have been considered in terms of potential for radiation processing [38,34], radiation modification has not been discussed in terms of foamed co-polymer blends that are the focus of this study. Both in terms of response to a specific dose of irradiation and more generally, these foamed vinyl nitrile polymers have distinct properties and behaviors from their solid counterparts, and utilizing the foam in quantitative engineering design, e.g. for impact protection, requires specialized engineering models. However, radiation processing-type effects remain unquantified, and studies, including those with subsequent development of structure-property relationships, often do not identify the potential effect of imaging radiation on the matrix polymer. Thus, the aims in this work are twofold: to highlight this potential issue for other researchers interested in combining *in-situ* mechanical testing with μCT imaging for polymeric materials, and to quantify and model the effects of this type of radiation exposure, i.e., X-ray irradiation, on a vinyl nitrile impact protection foam. To achieves these aims, the paper begins by describing a series of experiments used to determine the dose, baseline and modified polymer foam characteristics and properties using irradiation simulation, scanning electron microscopy, differential scanning calorimetry, dynamic mechanical analysis, Fourier transform infrared spectroscopy, and gel extraction. Based on the results of the mechanical testing, a viscoelastic material model with temperature and dose sensitive was formulated, implemented, and demonstrated as an open-source user element for finite element-based engineering design.

## 2. Materials and methods

**Fig. 2.**
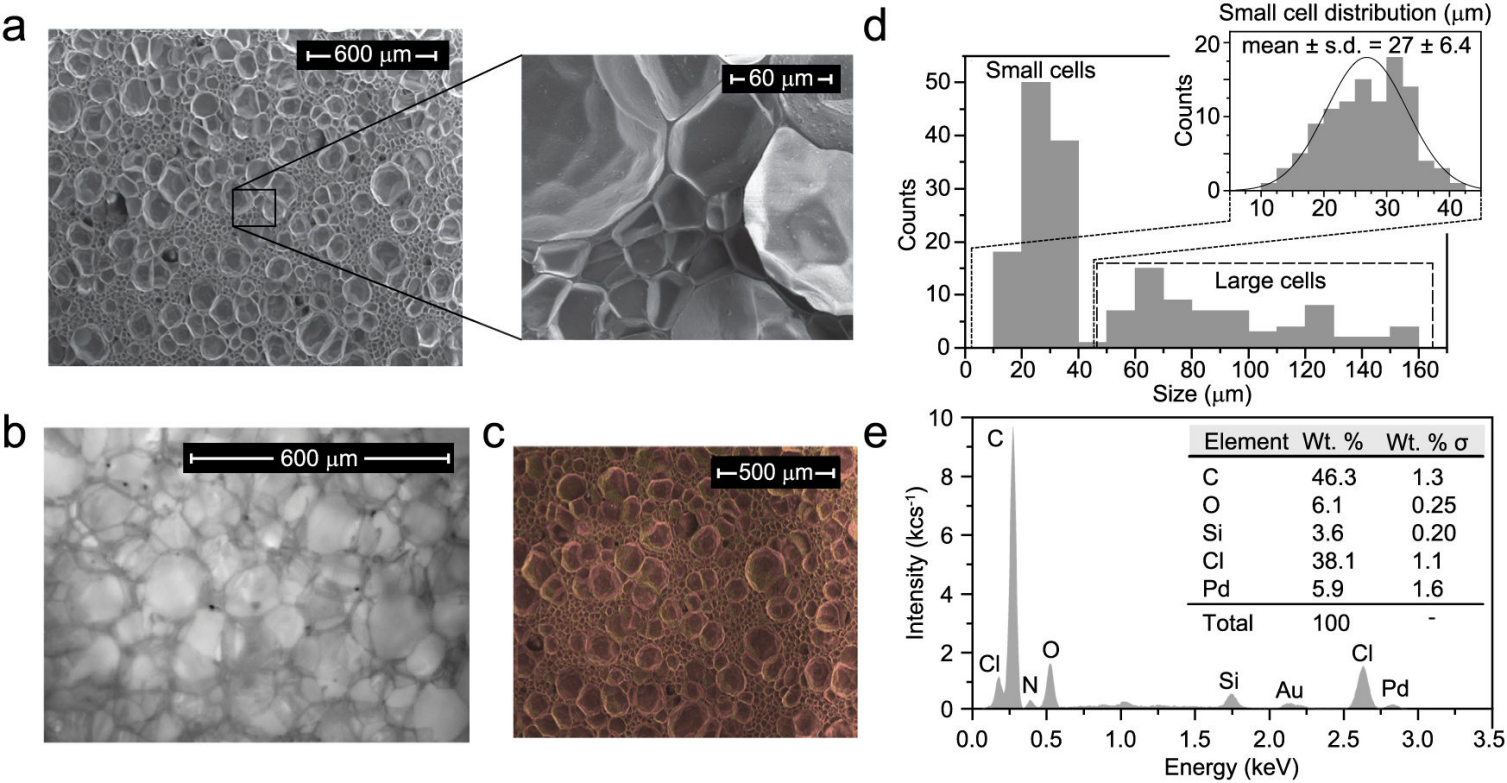
Scanning electron microscopy (SEM) with energy-dispersive spectroscopy (EDS) images and data, used to assess topology and elemental composition of the vinyl nitrile polymer foam. (a) SEM image of the foam surface, with inset showing a high-magnification view of several cells. (b) A comparable optical microscopy image of the foam surface (brightness and contrast adjusted to aid viewing). (c) EDS map of the foam, showing a relatively high spatial dispersion of elemental species. (d) Measurements of foam cell sizes from SEM, showing segregation between small and large cells. The inset shows the best-fit Gaussian to the small cell length distribution. (e) Spectral histogram of the EDS map showing signal intensity in thousands of detector counts per second (kc s^−1^) as a function of the emitted X-ray energy, with spectral peaks labeled. The inset table shows the approximate atomic weight percent composition estimate with 1-σ uncertainty from EDS.

Foam samples were excised from as-received sheets of an impact protection foam often used as a baseline for helmet liners and similar applications [17,40] consisting of a closed-cell matrix blend of vinyl nitrile polymer with a density of approximately 110kg/m^3^ (Impax VN600, Dertex Corp., Saco, DE^1^). Preliminary X-ray fluorescence analysis (X-Met 5100, Oxford Instruments America, Concord, MA; programmed to detect elements Mg through U) on fresh bulk material indicated Cl at an approximate mass fraction of 0.57 ± 0.006 and Zn at a mass fraction of 0.022 ± 0.0001, which was generally in agreement with the composition data obtained from scanning electron microscopy energy-dispersive spectroscopy (see Fig. 2), given that lower atomic number species were not measurable. Additionally, a sample was excised from the bulk material and prepared for scanning electron microscopy energy-dispersive spectroscopy (SEM EDS). Samples prepared for irradiation treatment were cylinders approximately 8.2 mm tall and 9.0 mm in diameter, which were excised from the bulk material with a cylindrical punch in a low-speed drill-press. Sample dimensions (height and diameter) were measured with three repeats using a digital caliper (resolution 0.01 mm). The samples were placed in an *in-situ* load frame at nominally 0 % engineering strain for approximately 1 d, 2 d and 3 d (all ± 0.5 h) for exposure to the X-ray tomography source. Exposure conditions were nominally set to 4 W and 50 kV with specimen-source working distance of about 24 mm and centered in the field of view for each treatment. Additionally, a sample was placed in a concentrated argon environment, transferred to a sealed argon-filled high-X-ray transmittance ampule and similarly irradiated for 3 d. These are compared to non-irradiated (0 d in-beam) – hereafter “fresh” – foams. These are typical imaging settings for previous structure-property experiments (hence placing specimens within the *in-situ* load frame without applying any load), and the specimens and magnification selected expose the entire specimen to a roughly spatially uniform beam intensity, as confirmed by placement of X-ray film at the specimen location. Vignetting leads to approximately 30 % lower exposure at the extremes of the corners, with the standard deviation in normalized intensity (detector counts) of between about ± 0.05 and ± 0.06 depending on the experiment.

### 2.1. Absorbed dose estimation

The absorbed dose to the foam was estimated based on Monte Carlo simulations of the irradiation process. The manufacturer of the CT system provided certain details of the spectral properties of the X-ray beam as well as the dose rate in air at 30 mm from the source. However, they did not provide details of the anode take-off angle, so all simulations were performed with 9°, 15°, and 21° angles and the variation due to this unknown factor was incorporated into the final uncertainties. As in the continuum scale mechanical response, the foam was assumed to be a uniform solid with a density of 0.11 g/cm^3^, which is equivalent to the average mass density of the material at the continuum scale. The assumption of uniformity does not cause a notable error in the computed average dose for low attenuation samples such as these foams [41]. Based on preliminary XRF data, the elemental composition of the foam was assumed to be H_144_C_98_Cl_42_N_2_Zn. The complete configuration, input, and output data from the simulation is available in the accompanying dataset (see section Data availability).

### 2.2. Specimen collection

Due to logistics and to ensure a stable baseline, two or more weeks post-irradiation samples within a given treatment category (number of samples N = 2) were randomly selected for either differential scanning calorimetry (DSC) (number of specimen repeats n = 2) and attenuated total reflectance Fourier transform infrared spectroscopy (FTIR-ATR, n = 3), or dynamic mechanical analysis (DMA, n = 1) and Soxhlet extraction (n = 3). Preliminary thermogravimetric analysis (TGA), stress relaxation, FTIR-ATR scans, DMA frequency and amplitude sweeps, and solvent extraction tests were used to evaluate the effect of radiation dose on foam properties, relevant details of which can be found in the supporting data. An initial cross-comparison of compression peak stress at a target strain level indicate good repeatability across *in-situ* load frame, universal test machine, and DMA uniaxial compression instruments. Despite the heterogeneous microstructure of the foam, the sample-to-sample variability at the continuum scale is low relative to the uncertainty budgets of each tests, which informed the decision to use only N = 2 samples. However, there were notable specimen-to-specimen differences post-irradiation in heat flow, likely attributable to non-uniformity of absorbed dose as a function of radial depth in the sample as discussed in 3.1.

### 2.3. Scanning electron microscopy with energy-dispersive X-ray spectroscopy

For SEM imaging, foam specimens approximately 3 mm by 4 mm were first sectioned from the sample to about 1 mm thickness, then mounted on an aluminum stub with carbon conductive tape, and finally sputter coated with a 3 nm gold-palladium (Au-Pd) conductive coating for optimal imaging conditions. The surface morphology of the specimens, including the size of the foam cells and ligament thicknesses, were imaged on a FEI Helios NanoLab 650 dual-beam SEM equipped with an X-ray detector that allows for elemental mapping with EDS. The micrographs and spectra of the specimens were collected under high vacuum, i.e., less than 0.4 mPa (3 × 10^−6^ torr), at a beam energy of 5keV and probe current between 0.1 pA and 0.5 pA, with scan parameters optimized for clear images with minimal charging, artifacts, and drift. For comparison, an extended depth of field brightfield optical microscope image of the foam surface was also collected (Zeiss Axiovert 200 M) using a 50-image stack at 1.5 μm with approximately 1.024 μm per pixel resolution. To measure cell size, four diameter measurements were made in ImageJ/FIJI [42] for each complete, non-occluded cell in the field of view using the length scale reference embedded in the image by the instrument. The mean and standard deviation was recorded for each cell. The cell wall (i.e., ligament) thickness was measured similarly, with one measurement per feature and recording the overall mean, standard deviation, and standard error.

### 2.4. Differential scanning calorimetry

For the DSC measurements, samples were sectioned such that specimens of 3 g to 5 g from near the geometric centroid region of the cylinder were harvested. The specimens and aluminum hermetic pan were weighed to ±0.1 mg and specimens were sealed into the pans. Two specimens were collected from each sample. Each specimen was measured over three heat-cool temperature sweeps in a nitrogen environment between −80 °C and 100 °C at approximately 10 °C/min with a 10 min temperature equilibration between segments. A midpoint inflection analysis between −40 °C and 60 °C was used to determine the glass transition temperature T_*g*_ in the second and third heating cycles. To determine if differences from the fresh sample were significant, the T_*g*_ measurements were compared using heteroscedastic paired *t*-tests at a significance level of *p* < 0.005.

### 2.5. Dynamic mechanical analysis

For the DMA measurements, one full specimen for each treatment duration was used in a separate actuator-transducer-type instrument. Specimens were fixed to stainless steel flat platens with a thin layer of quick-setting epoxy resin. A preliminary study determined that interfacial properties of the epoxy bond had no noticeable effect on the modulus measurements and eliminated potential artifacts due to loss of contact at low temperatures or high frequencies. Additional preliminary studies included the stress relaxation at approximately 30 °C (a circa 7.9 s strain step at about 76.28 %/min followed by a hold for more than 900 s), as shown in Fig. 1b, and several amplitude and frequency experiments to determine the linear viscoelastic response envelope for the material (e.g., see data presented in [35]). The DMA probed the linear-elastic response region of the foam polymer matrix using a small-amplitude oscillatory strain approach for a fixed frequency of 10 rad/s over series of temperatures, roughly 60 °C to −30 °C in 5°C increments. Several tests that were conduct at below the glass transition temperature (T_*g*_) of the polymer (ca. −20 °C or less) were unable to be completed due to excessive force relative to machine limits for minimum strain and maximum force. At each temperature the specimens were allowed to equilibrate to a low compressive force level of about 0.05 N for at least six minutes to account for thermal strain, which is significant (*p* < 0.005) throughout the temperature range and non-linear about the T_*g*_. At each temperature, 10 measurements were taken over 120 s from the imposed sinusoidal strain ($\epsilon =\epsilon_{0}\;\sin\;(\omega t)$, where $\epsilon_{0}$ is the strain amplitude, ω is the frequency, and *t* is time) and measured stress ($\sigma =\sigma_{0}\;\sin\;(\omega t+\delta )$, with δ the phase lag between strain and stress). Measurands are the storage modulus $E^{\prime }=\frac{\sigma_{0}}{\epsilon_{0}}\;\cos(\delta )$, loss modulus $E^{\prime \prime }=\frac{\sigma_{0}}{\epsilon_{0}}\;\sin(\delta )$, and loss tangent (tan δ, also called the dissipation factor). Between each temperature step the polymer was allowed to recover with axial force less than ∣0.05 N∣ at approximately 60 °C for 1.5 h or longer to remove any thermal history effects. Tests were configured to automatically adjust strain and axial load levels to remain above operational noise floors of the instrument and below the onset of the plateau regime of the foam. The standard uncertainty on the frequency and moduli is estimated to be ± 5 % and ± 8 %, respectively and the standard uncertainty in the temperature is ± 0.1 °C. To determine if differences in mechanical properties at 20 °C were significant, the measurements were compared using a heteroscedastic paired *t*-test at a significance level of *p* < 0.005.

### 2.6. Fourier transform infrared spectroscopy

For the FTIR-ATR measurements, unused material from the same fresh and 3-day treatment condition samples used for DSC measurements was sectioned into thin specimens (about 2 mm) from near the geometric centroid. Specimens were clamped in the ATR arm with consistent clamping force for all specimens. No noticeable differences were observed with small variations in clamping force, specimen thickness, or tip geometry (flat vs. concave). All ATR data were collected on a calibrated commercial instrument (Nicolet iS50 FTIR, Thermo Fisher Scientific Inc., Madison, WI) with a built-in diamond ATR module, a monolithic diamond ATR crystal, and a temperature controlled DLaTGS detector (5000 cm^−1^ to 100 cm^−1^). Before each run a background spectrum was taken and automatic background subtraction was applied to the corresponding spectrum measurement. All spectra represent an average of 128 scans using a 4 cm^−1^ resolution, from approximately 570 cm^−1^ to 3870 cm^−1^. Typical standard uncertainties for the spectral measurement are 4 cm^−1^ for wavenumber and 5 % in peak intensity measurements.

### 2.7. Gel content

The gel content of fresh and 3-day irradiated vinyl nitrile foam specimens sectioned from DMA specimens was measured via Soxhlet extraction. Gel content is a measure of the extent of insoluble material in the polymer, indicative of the crosslinking density. The higher the gel content, the higher the crosslinking density of the specimen. The initial mass of each specimen was measured ($m_{i}\approx 3$ mg) with an accuracy of ± 0.01 mg, and then each specimen was placed into a fiberglass thimble. The extraction was conducted at approximately 70 °C in 200 mL of a 50/50 (vol/vol) mixture of tetrahydrofuran-dichloromethane (THF-DCM) until equilibrium for 48 h or more. The final mass ($m_{f}$) of each dried sample was recorded and used to calculate the gel content based on the equation: $Gel\;content\;(\%)=100\times \frac{m_{f}}{m_{i}}$. At least three samples per condition were used to calculate the gel content and the reported numbers represent mean ± Standard Error of Measurement (N ≥ 3). The propagated uncertainty on gel content is ± 0.5 %.

## 3. Results and discussion

The specific damage processes of ionizing radiation on acrylontrile-butadiene-based rubbers and poly(vinyl chloride) polymers – the matrix polymer blend of the foam in this study – are outside the scope of the present study. However, besides the crosslinking and chain scission processes mentioned above, radiation exposure has been linked to oxidization and yellowing of the polymers, and, thus, antioxidant additives are often used [43,34]. In the case of this vinyl nitrile foam, visual inspection of irradiated specimens reveals notable yellowing, particularly for higher doses and specimens irradiated in the presence of oxygen. Specifically, upon comparing a sectioned sample from the argon-environment treatment and the atmospheric treatments, in the argon-treatment a more notable color gradient exists from the exterior surface to the core (white-to-yellow). Since this is a closed cell foam argon permeation will be limited to diffusion through the polymer matrix and cell membrane walls to reach the interior void space. Since the yellowing effect is not seen in the air-treatment is likely that it is due to a gradient in available oxygen, rather than depth-dependency of the absorbed dose. Preliminary evidence of a crosslinking mechanism driving mechanical changes, rather than oxidative processes as in the yellowing, was observed following *in-situ* compression experiments - the applied deformation was permanently set into the material following the radiation exposure for 3D tomographic imaging, whereas no permanent set is observed in conventional universal testing system experiments to similar or greater compressive strain levels.

### 3.1. Absorbed dose estimation

Based on the results of the absorbed dose simulations, it was determined that the average dose to the foam was (8.1 ± 0.9) kGy per day. The dose was not deposited uniformly in the foam, with the core of the foam receiving a lower dose than the radial exterior due to attenuation of the x-ray beam by the foam (approximately 5.5 kGy/day near the center of the foam vs approximately 12 kGy/day near the surface), with a non-linear decay in absorbed dose as a function of radial distance from the center. The variation in dose as a function of specimen height was below the noise floor of the simulation. The complete modeling results are available in the data repository (see section Data availability). These results for absorbed dose variation informed the sample dissection and specimens collection process for the DSC, FTIR, and Soxhlet.

### 3.2. Scanning electron microscopy with energy-dispersive spectroscopy

#### Scanning electron microscopy

SEM micrographs (Fig. 2) of a sectioned surface of fresh foam revealed the presence of roughly equiaxed irregular spheroidal cells with various sizes, consistent with previously reported μCT imaging of these foams [14]. There is a distinguishable difference between a population of smaller cells (approximate diameters of 10 μm to 42 μm), and larger cells with diameter between approximately 55 μm and 155 μm. The small cell population broadly follows a skewed normal distribution with an average cell diameter of (26.8 ± 6.40) μm (Fig. 2), whereas the larger cells have a wider spread of sizes, (74.4 ± 13.8) μm, and a longer tail. In addition, the cell wall thickness is straightforwardly measurable in the higher magnification images, see Fig. 2c, and were (0.95 ± 0.5) μm, which agrees well with auto-correlation function-based estimates from μCT images [44]. This micrograph is comparable to the extended depth of field optical image in Fig. 2b, although the brightfield optical microscopy is unable to clearly resolve the small-size cell population.

#### Energy-dispersive spectroscopy

EDS analysis elemental mapping showed the presence of carbon (C), chlorine (Cl), nitrogen (N), and oxygen (O) elemental species in the vinyl nitrile, with relatively uniform dispersion throughout the specimen (see Fig. 2c). The full EDS spectrum from the map is shown in Fig. 2e. The C atoms are part of the backbone of both polymers, while the N atoms are characteristic of the nitrile rubber, and the Cl atoms are solely present in the chemical structure of polyvinyl chloride (PVC). Oxygen atoms are due to oxygen molecules in the gaseous phase of the biphasic foam and may be also associated with certain oxidation species, as was identified in FTIR spectroscopy (see peaks at 1715 cm^−1^, 1109 cm^−1^, and 3390 cm^−1^ in Fig. 5c). The smaller intensity peaks of gold (Au) and palladium (Pd) are present due to the 3 nm conductive surface coating. A small feature at approximately 1 keV may be associated with L-*α* emission from the trace quantities of zinc identified by XRF, but was an insufficiently distinct peak to be included in the composition analysis. Finally, trace amounts of silicon (Si) were present and determined to be contamination from adjacent mesoporous silica samples (fine powder) during the sputtering and imaging processes. The percent by weight composition data are shown in the inset table of Fig. 2e.

### 3.3. Differential scanning calorimetry

**Fig. 3.**
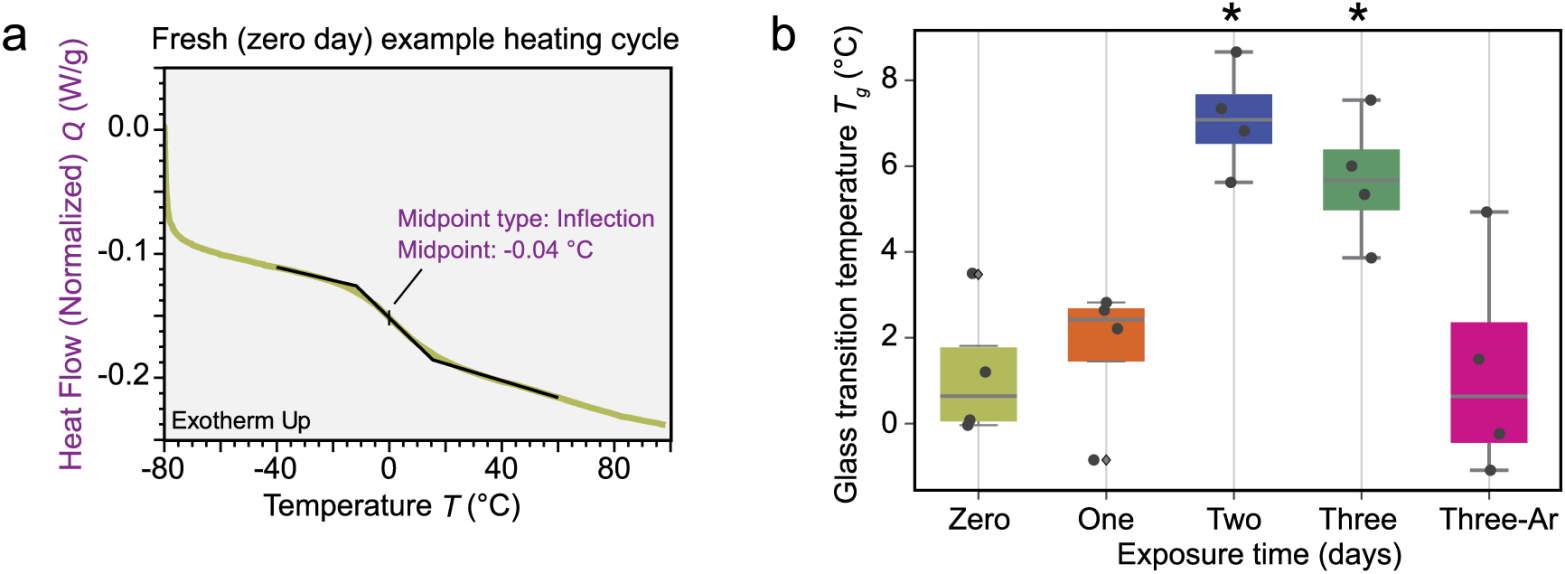
Example heating curve and processed DSC data for the foam. (a) Example heating cycle showing the inflection mid-point fitting used to compute the glass transition temperature (T_*g*_) of the polymer matrix material. (b) Complete T_*g*_ results across each treatment condition. The 3-day-Ar indicates the argon-rich experiment, as described in the Methods section. Starred columns (viz. 2 day and 3 day) indicate a significant (heteroscedastic paired *t*-test, p < 0.005) deviation from the fresh case. Standard uncertainties associated with the use of DSC in the measurement of these thermal properties are approximately 5 %.

The DSC data consisted of normalized heat flow measured as a function of temperature for a fixed heating rate. The raw data curves showed small, relatively broad glass transitions in the heating cycle between about −20 °C and 30 °C. No other salient features were observed. The midpoint of the glass transition region was recorded as described in the Methods, and occurred at approximately 0 °C for this example from the fresh case. A representative example is given in Fig. 3a, which includes the midpoint fit used to determine the T_*g*_. These general features of the raw data remain similar between treatments.

The analyzed results, i.e., the T_*g*_ measurements for each sample and heating cycle, are shown as a box plot with individual datapoints overlayed in Fig. 3 for each treatment condition. The mean glass transition temperature of the baseline foam is approximately 1 °C, but measurements ranged from −0.04 °C to 3.5 °C. There was a small, but not significant (*p* < 0.01), increase in mean T_*g*_ after one day of exposure (approximately 8.1 kGy). After two days and three days of exposure there was a significant (*p* > 0.01), although still relatively small, increase in T_*g*_. Since the DSC measurements are made on small material volumes sampled from the larger specimens, some variability between specimens is likely driven by the radial dose-dependence identified by the dosage modeling. The final treatment, three days in an oxygen-poor, argon-rich environment resulted in no significant change (*p* < 0.01) in mean T_*g*_ from the fresh foam, but a notable increase in scatter of the T_*g*_ data over the range observed in under other treatment conditions. The increased scatter in the T_*g*_ is likely due to the argon diffusion artifacts of the conditioning and sample procedure described above. Although care is taken to consistently excise samples, if an argon diffusion gradient corresponds to a property gradient, variability in the properties of the sectioned cores for DSC would be expected to increase.

### 3.4. Dynamic mechanical analysis

**Fig. 4.**
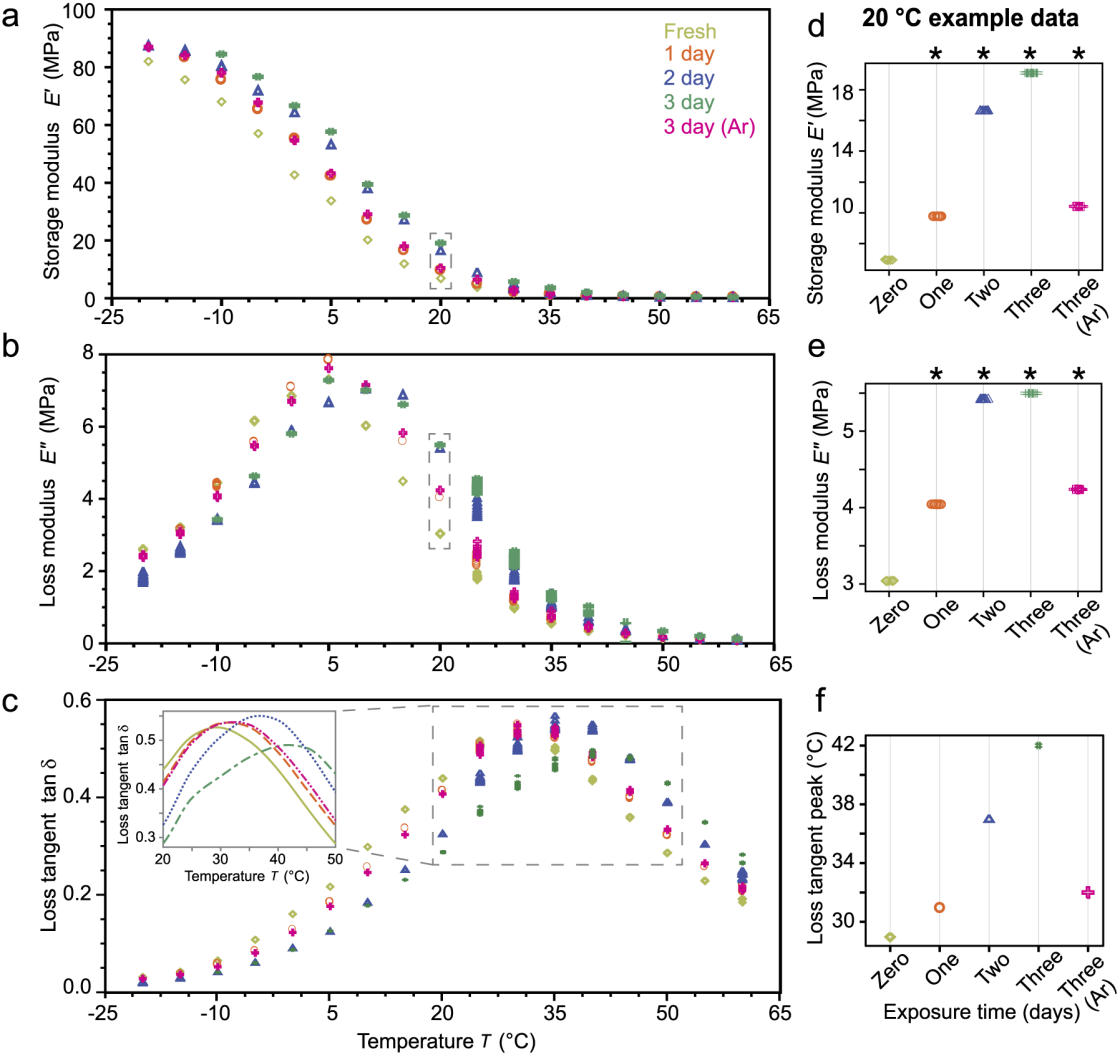
Storage modulus, loss modulus, and loss tangent data for the foam across a range of temperatures and treatment conditions. (a) Storage modulus (E′) a function of temperature from the onset of glassy behavior at circa −20 °C into the rubber behavior regime at up to 60 °C. (b) Loss modulus (E″) over the same temperatures as in (a). (c) Loss tangent over the same temperature range. The *inset* shows interpolated mean tan δ versus temperature for each treatment condition, to highlight changes in peak tan δ temperature. The storage modulus (d), loss modulus (e) 20 °C (dashed boxes in (a), (b)) highlighting the room-temperature changes in properties. Starred columns indicated significant (heteroscedastic paired *t*-test, p < 0.001) differences from the baseline (zero-day). (f) Change in loss tangent peak (roughly equivalent to the glass transition temperature) for increasing exposures, based on interpolation of the data (see *Inset*).

Data from the DMA experiments consist of storage modulus, loss modulus, and loss tangent as a function of temperature from the glassy regime to the rubbery regime of the polymer matrix material, for each treatment condition. Plots of these data are given in Fig. 4 and complete data is available in the supporting data.

#### 3.4.1. Storage modulus

Storage modulus results show a glassy-to-rubbery-type transition with a gradual decrease in stiffness beginning at around −20 °C and 82 MPa and continuing until a plateau at approximately 40 °C and approximately 0.7 MPa for the fresh foam. The storage modulus increases and the transition region moves to higher temperature with increased X-ray exposure. The transition region of the 3 d exposure begins at around −15 °C and 86 MPa and plateauing at approximately 40 °C and 2 MPa. The complete data are shown in Fig. 4a.

Examining the behavior at a constant temperature of 20 °C more closely, Fig. 4d shows that exposure to X-rays significantly increased stiffness compared to the fresh foam (*p* < 0.001), with changes between 0 d and 1 d exposures and between 2 d and 3 d exposures were slightly smaller than change between 1 d and 2 d exposures. The 3 d argon environment case significantly (*p* < 0.001) reduced the rate of change in stiffness compared to an equivalent 3 d exposure in air atmosphere, and was roughly equivalent to the 1 d (air) exposure case. The change in stiffness at 15 °C and 20 °C is about a factor of 3×, which increases to a factor of over 4× at 25 °C and about 3.5× at 30 °C. Changes in storage modulus are less noticeable in the rubbery plateau region, around a factor of 1.5×.

#### 3.4.2. Loss modulus

The effect on loss modulus is similar to storage modulus; an initial increase in magnitude (for the 1 d treatment) followed by a shift of the peak toward higher temperature (rightward), see Fig. 4b. The loss modulus shifted upward, indicating increased dissipation, by a factor of about 1.5× to 2× at 20 °C for the 2 d and 3 d treatments. The change in absolute peak of the loss modulus was relatively small (ca. 7 % increase, which occurs at approximately 5 °C for the 0 d and 1 d treatments, and increases to about 15 °C for the 3 d treatment. It is noted the 2 d magnitude is similar to the 3 d.

Comparing the treatment conditions at 20 °C, see Fig. 4e, reveals a significant increase in dissipation over baseline (*p* < 0.001). A substantial increase in dissipation was measured during the first two days of exposure, and a smaller increase from 2 d to 3 d. This is consistent with the DSC measurement of changes in T_*g*_. Again, the 3 d argon treatment condition seems to be similar to the 1 d air treatment condition.

#### 3.4.3. Loss tangent

The loss tangent as a function of temperature for each treatment condition is shown in Fig. 4c. The magnitude of the peak tan δ between the treatments varies a relatively small amount – all treatments were in the range tan δ=[0.49,0.55], inclusive). The differences in tan δ in the rubbery regime remain more apparent than in moduli data, although they are less apparent in the glassy regime where tan δ is below about 0.10. The curve shifts toward high temperatures, as was also observed in the loss modulus. As in the storage and loss moduli, the 3d-argon treatment is quite similar to, and often overlapping, the 1 d air treatment.

To better visualize the temperature shift, the inset to Fig. 4c shows the peak regions in tan δ interpolated with a Makima interpolating function sampled at 0.05 °C increments and plotted as continuous lines. The temperature at which the maxima occur, associated with the T_*g*_ of the polymer, shifts toward higher temperature. This increase in T_*g*_ is consistent with the measurements made with DSC, shown in Fig. 4b. To quantitatively highlight this, the estimated peak temperature (approximated as the maximum of the interpolant) is plotted against treatment condition. In agreement with the DSC measurement of T_*g*_ changes, different in peak temperature from 1 d to 2 d is slightly larger than the other changes, although the overall trend in peak temperature appears to be broadly linear with exposure time.

Image-based modeling and *in-situ* analysis of foam, i.e., meso-scale analyses, are important methods to gain insight into foam behavior. These often rely on material properties, such as linear elastic stiffness, to measure or predict key deformation features such as hinging and buckling, which govern critical continuum-scale features such as plateau stress (i.e., ligament buckling) and apparent linear elastic modulus [45-47,16]. Relatively minor increases in matrix stiffness, such as was observed at the 1 d dosage, have been shown to substantially affect critical load and plateau stress, e.g., for closed cell low density polyethylene foam models [20]. Thus, meso-scale analyses informed by serial *in-situ* μCT imaging (e.g., volumetric images at increasing strain levels, or to monitor a phenomenon) should consider the potential for changes to the matrix material properties, and resultant alteration of deformation mechanisms, as a consequence of the imaging protocol. Furthermore, rate-dependent viscous effects have been investigated in terms of continuum responses, for example [4,48,49,40], or via micro- and meso-structural effects and rate dependency as in [50-53], among others. The contribution of viscoelastic effects to meso-scale mechanics, i.e., the aforementioned hinging and buckling mechanics, is less well studied than those of stiffness and plasticity, although studies such as [54] and [55] discuss viscous contributions to analyses that incorporate micro- and meso-scale deformation mechanisms. Thus, as with stiffness, unexpected changes in dissipation have the potential to confound experimental and modeling efforts, but may be a potential tool for material designers.

### 3.5. Fourier transformed infrared spectroscopy

**Fig. 5.**
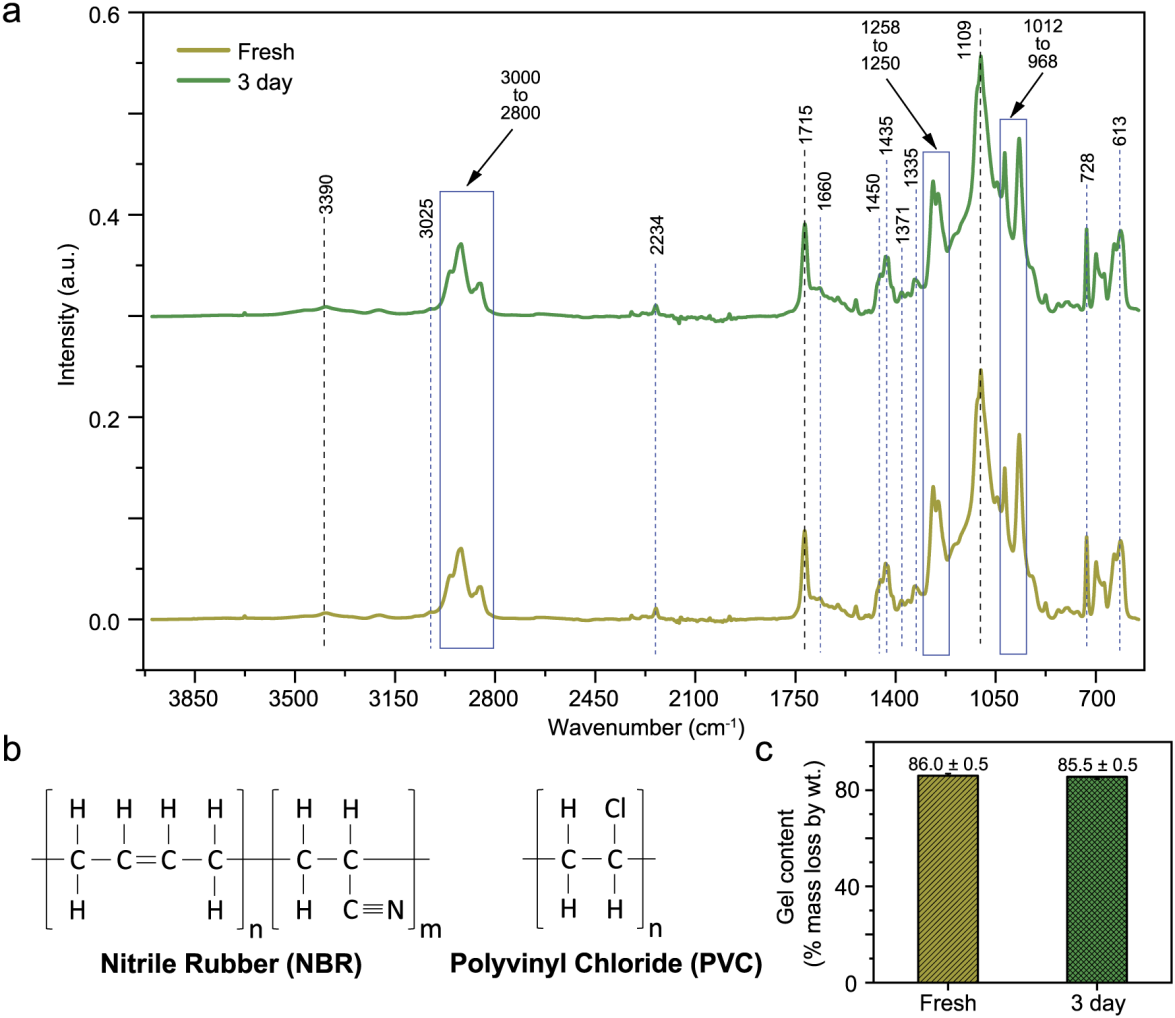
Polymer structure and structural change assay data from FTIR-ATR and gel extraction on fresh and the longest, 3-day irradiated treatment. (a) FTIR-ATR spectra data with salient peaks and regions labeled with their specific wavenumbers. The associated molecular structures are described in the text. The peaks are consistent with a partially crosslinked blend of PVC and NBR. (b) An illustration of the monomer unit structure of the NBR (left) and PVC (right) to guide the reader in interpretation of the spectral peaks. (c) Gel content measured using the Soxhlet extraction process from fresh and 3-day irradiated foam specimens, shown as bar plots with error bars of one standard error from n = 3 specimens.

The FTIR-ATR spectra are shown in Fig. 5a for the blended polymer foam. The monomer unit structures for both polymers in the vinyl nitrile blend are shown in Fig. 5b, and the spectra are consistent with a blend of this nature. The spectra show a small absorption band at 3025 cm^−1^, assigned to sp^2^ hybridized = C─H bond stretching. This is caused by the presence of carbon─carbon double bonds in the NBR material [56-58]. The stronger characteristic absorptions in the region of 2800 cm^−1^ to 3000 cm^−1^ are attributed to the stretching of sp^3^ hybridized C─H bonds (2981 cm^−1^) and asymmetric stretching of (CH_2_) methylene groups (2919 cm^−1^ and 2852 cm^−1^) in both the NBR and PVC materials [56-58]. The small absorption band observed at 2234 cm^−1^ is attributed to the stretching of the C≡N group, characteristic of the NBR polymer [58]. Also, the absorption band at 1660 cm^−1^ is attributed to the stretching of C ═ C in the NBR [58]. The peaks at 1450 cm^−1^ and 1435 cm^−1^ are related to bending of C─H groups and asymmetric bending of (CH_3_) methyl groups, as well as the smaller band at 1371 cm^−1^ associated with rocking of methyl groups [56-58]. The absorption band at 1335 cm^−1^ is assigned to CH_2_ deformation [58]. The next two strong absorption bands at 1258 cm^−1^ and 1250 cm^−1^ represent the stretching of C─H groups from Cl─CH bonds (characteristic of the PVC) and the rocking mode of C─H groups respectively [56,57]. The next two absorption bands at 1012 cm^−1^ and 968 cm^−1^ are assigned to wagging bending vibration of vinyl and trans C ═ C groups [58]. The peak at 728 cm^−1^ is assigned to rocking of methyl groups usually present in long chain alkanes and the last peak at 613 cm^−1^ is characteristic to the PVC material and is assigned to C─Cl stretching [56-58]. Finally, the two strong absorption peaks at 1715 cm^−1^ and 1109 cm^−1^ are indicative of oxidation in the material and are assigned to C ═ O stretching of carbonyl groups and stretching vibration of C─O bonds respectively [56,27]. The evidence of oxidation in the material is further supported by the small and broad peak at 3390 cm^−1^ attributed to ─OH stretching [57].

The IR spectra of the fresh and 3-day irradiated foams show no evolving peaks. Both spectra include evidence of oxidation present in the material. It is worth mentioning that the fresh materials can be oxidized as oxygen gets trapped within the closed cells of the foam during the manufacturing process (extrusion), while the material undergoes mechanical stress at high temperatures. There is no clear difference in the oxidation peaks between the irradiated and fresh samples, suggesting that the irradiation process did not induce detectable oxidation changes to the foams.

### 3.6. Gel content

The results of the gel content (% by weight) of fresh and 3-day irradiated foam specimens are shown in Fig. 5c. As mentioned above, the gel fraction is directly proportional to the insoluble material in the polymer; therefore, a higher gel fraction indicates a higher degree of crosslinking. The results indicate that there is no detectable change (p = 0.105) in the crosslink density between the fresh (86.0 % ± 0.2 %) and the 3-day irradiated (85.5 % ± 0.2 %) foam samples. Given the degree of sample variability and level of uncertainty in the gel content measurement, a small percentage point change in the crosslinking, which can notably change the mechanical properties, might be below the detection limit of the extraction method.

### 3.7. Polymer irradiation mechanisms

**Fig. 6.**
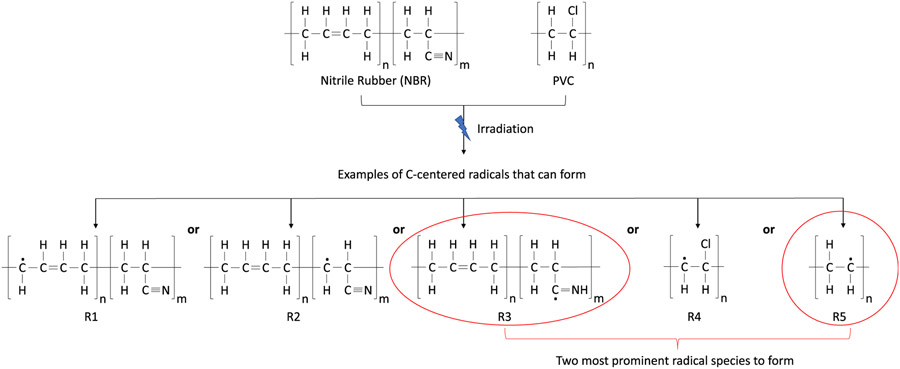
Likely C-centered radical species formed during irradiation of vinyl nitrile materials. There are five radicals possible, labeled from R1 to R5, but R3 and R5 are expected to be the most favored. The acrylonitrile and vinyl-chloride are most susceptible. It is noted the dosage levels in this study are mild compared to typical radiation processing or damage studies.

The vinyl nitrile viscoelastic and thermal properties were the most sensitive to irradiation. Complementary chemical spectroscopy and structural measurements of irradiation damage were inconclusive and likely hampered by the challenges of a closed cell, heterogeneous structure. This section gives a brief review of the damage pathways expected for the vinyl nitrile foam. This provides a qualitative method to discuss whether the measured changes in thermophysical properties are consistent with more routine radiation processing, especially given the lack of detailed formulation knowledge about the commercial material and radiation source. As discussed earlier, the vinyl nitrile foam is a blend of polyacrylonitrile and polybutadiene rubber and polyvinyl chloride. The foam is initially crosslinked during manufacturing. During the irradiation of nitrile rubber materials (NBR), carbon center radicals can form in both the polyacrylonitrile and polybutadiene segments of the copolymer through hydrogen abstraction. In the case of polyacrylonitrile, hydrogen abstraction can result in the formation of alkyl and allyl radicals, shown as R1 in Fig. 6. Hill et al. [59] confirmed the presence of these radicals via electron paramagnetic resonance (EPR) spectroscopy after gamma-irradiation of nitrile rubber samples at a dose rate of 3 kGy/h. Also, hydrogen abstraction from the methylene group and radical addition to the nitrile group of the polybutadiene was also observed, resulting in radicals R2 and R3 as shown in Fig. 6 [59]. The radiation chemical yield of radicals in polyacrylonitrile is greater than for polybutadiene, which means that a higher fraction of radicals could potentially be formed on the acrylonitrile groups [59]. Furthermore, allyl carbon center radicals are more thermally stable than alkyl radical because of resonance effects [59,60]. Therefore, carbon centered radicals formed on the acrylonitrile unit of the copolymer can abstract hydrogen from butadiene units to form additional allyl radicals. These radicals could be located either on the same polymer chain or on neighboring polymer chains.

The irradiation effects on polyvinyl chloride (PVC) include cleavage of an H (radical R4 in Fig. 6) or a Cl (radical R5 in Fig. 6) atom from the main polymer chain, which can lead to the formation of carbon center radicals. However, it has been demonstrated that the primary radical formed will result from the abstraction of a chlorine atom and the cleavage of a C─Cl bond, as shown in Fig. 6 [61]. The chlorine atom can then abstract other hydrogen atoms from adjacent polymer chains of PVC or even NBR units to form new unstable carbon center radicals [25]. This process is generally described as a radical propagation reaction in radiation chemistry, and leads to the formation of hydrogen chloride (HCl) gas and conjugated unsaturation (long polyene units) [62,63], see again Fig. 6. All of the components in the vinyl nitrile foam are known to form carbon centered radicals under the relatively mild exposure conditions of the X-ray. The resulting carbon centered radicals in the PVC and NBR units of the vinyl nitrile foam samples can then further participate in crosslinking reactions (termination reactions) through radical-radical recombination between C-centered radicals in adjacent polymer chains. The addition of mild crosslinks into a polymer system results in increases in stiffness and the glass transition temperature, consistent with those shown in Fig. 4. However, in the presence of oxygen, C-centered radicals can react with O_2_ within the material and form peroxy radicals, which can then abstract hydrogen atoms from a neighboring molecule to form hydroperoxide groups or combine with other available radicals to from peroxide groups (termination reaction). Hydroperoxides are not thermally stable and at temperatures above about 70 °C can decompose (propagation reaction) to form alkoxy radicals and hydroxyl radicals, which are very reactive radical species that will degrade the material through main chain C─C scissions [21,26,27]. There is likely a combination of crosslinking and oxidization processes, as evidenced by differences in the Ar-environment experiments, smaller changes between the 2 d and 3 d time frames, decrease in tanδ magnitude at 3 d, and the longer relaxation times in the stress relaxation experiments.

## 4. Linear viscoelastic material model

Vinyl nitrile foams are utilized in impact protection applications where engineering optimization of the padding is an important component of the system performance. To model and design with the effects of the viscoelastic property changes radiation exposure a linear viscoelastic finite element (FE) user material has been developed that includes both temperature and dose as model parameters. For similar viscoelastic materials, several approaches have been taken, for example, fractional derivative-based fitting [64-66], generalized Maxwell models with non-linear elements for large deformations [4], and modifications of the multi-parameter Odgen-type model (the so-called “Hyperfoam”) [67,39].

### 4.1. Material model and finite element setup

A standard linear solid (SLS) material model is used, similarly to the model framework for the design methodology proposed by Rahimzadeh et al. [68]. The model includes input terms for temperature- and dose-based changes in the rate-dependent constitutive properties of the material. Calibration data from small-amplitude oscillatory strain for these changes are analytically mapped to the material parameters of the SLS. A three dimensional model framework is adopted based on the implementation discussed by Kaliske and Rothert [69]. The theory and implementation details are provided in Appendix A. In brief, the 3D SLS model is discretized in a time-implicit finite element formulation, which involves four material properties that completely parameterize the SLS. These are Poisson’s ratio (ν), rate-independent elastic modulus (the main spring, $E_{0}$), relaxation time ($\tau_{1}$), and normalized rate-dependant elastic modulus ($\gamma_{1}$). Poisson’s ratio in similar materials is primarily a function of mesostructure in the small-strain regime [19], and it is thus assumed to take a constant value at ν=0.23 from its mean value between 0.1 % and 5.1 % axial engineering strain in reference data [35]. The stiffness of the main spring was assumed vary in proportion to radiation dosage only, i.e., $E_{0}(D)$, and was computed from the measured stress and strain at the end of stress relaxation experiments (shown in truncated, normalized form in Fig. 1d) as $E_{0}={[\sigma (t)∕\epsilon (t)]\;|}_{t=1600\;s}$. The mean of the storage and loss moduli measurements (see Fig. 4), were used to compute $\gamma_{1}$ and $\tau_{1}$ via $\gamma_{1}=\left(E^{\prime }∕E_{0})(1+\omega^{2}\tau^{2}\right)∕(\omega^{2}\tau^{2})$ and $\tau_{1}=1∕(\omega \tan\;\delta )$ for each dose and temperature combination. The open-source user material model (UMAT) that includes specialization and calibration for temperature and dose dependant material properties was written in FORTRAN as a traditional UMAT subroutine suitable for Abaqus Standard or CalculiX. The complete open source implementation is included in the data for this paper (see section Data availability) and available via GitHub.

### 4.2. Finite element validation and demonstration

**Fig. 7.**
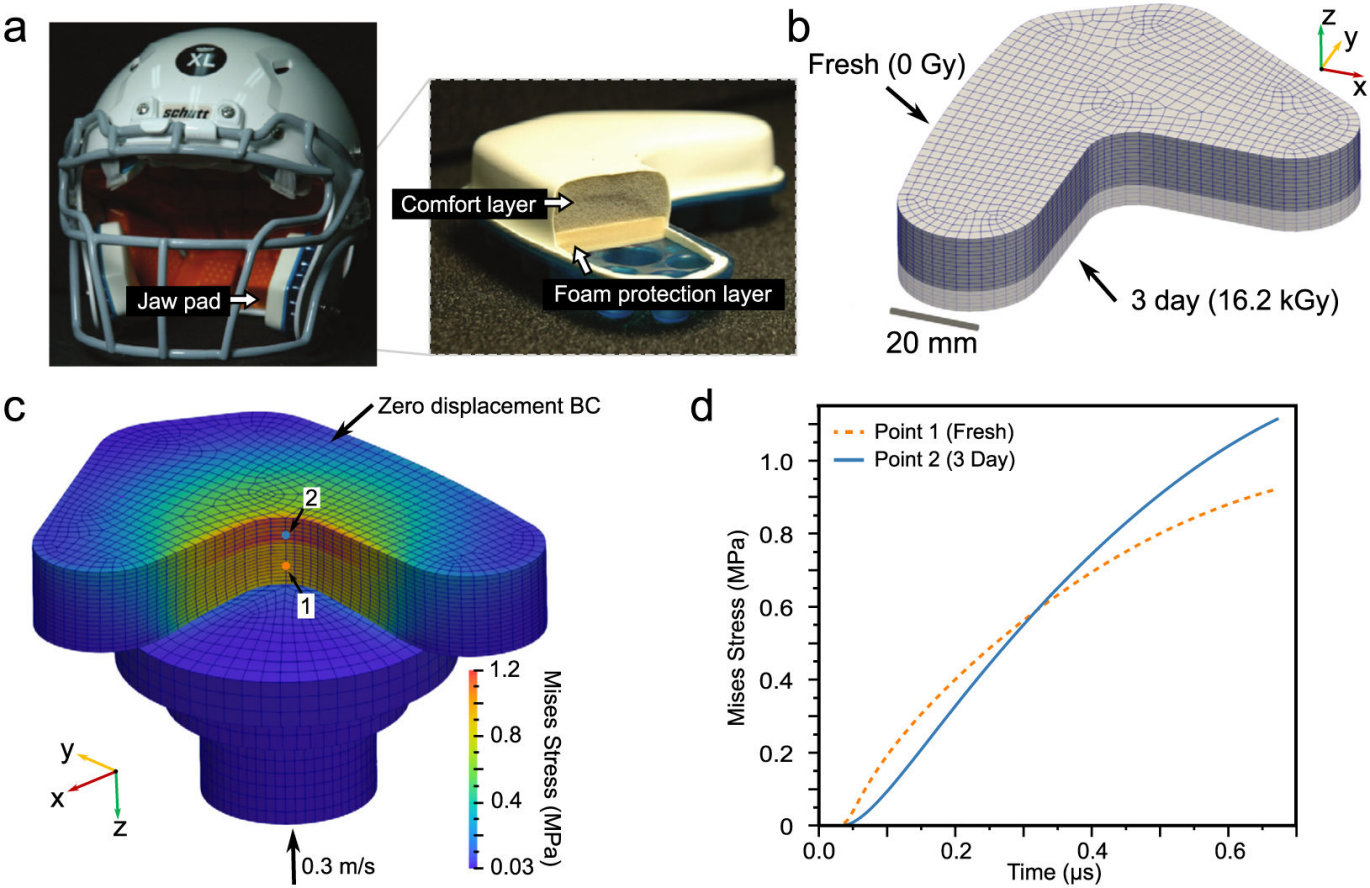
An example of foam response engineering using X-ray processing for impact protection. (a) youth American Football helmet, and cutaway of foam liner showing soft (“comfort”) and stiff “protection”) foam layers. (b) Simplified example pad geometry to demonstrate radiation processing design using this UMAT implementation, with two regions of different applied radiation dose; (c) impact simulation results, showing stress segregation between irradiated and fresh material. (d) Stress versus time traces for two nodes in the two different regions, as indicated by the color-coded points in (c).

The FE model was first validated through direct comparison of a virtual DMA test with the DMA experiments outlined above, see sub-section Appendix A.3. To demonstrate the applicability of the FE model in a practical application, a simplified American Football helmet pad, used for impact mitigation, was modeled. The pad generally takes the form of two layers of dissimilar materials, one stiffer and one softer (Fig. 7a, adapted with permission from [14]). Radiation processing was applied to the geometry representative of this pad, to create a single pad with two regions: one with which has the base properties (0 Gy) and one with 16200 Gy of radiation dose, applied via the material model (Fig. 7b). A constant temperature of 20 °C was applied. An impact was simulated on the material, using a standardized impactor form [70] and impact velocity of 0.3 m/s. Segregation of stresses achieved, whereby peak stress and stress rate have been mitigated directly below the impactor (Fig. 7c-d). Although this bi-layer design is a basic example, it showcases that radiation modification coupled with mechanical modeling provides a non-invasive mechanism to design and fabricate useful structures to alter mechanical response. There is a wealth of mechanics research representing many potential applications for the design of heterogeneous stiffness and rate dependent properties (i.e., custom metamaterials). Functional gradients are an important design consideration for reducing injury risk across a broad range of impact energies. The ability to utilize ionizing radiation to fabricate functional gradients in this foam may open new design space for protective material designs.

## 5. Conclusions

X-ray-based processing of polymers, including crosslinking, chain scission and other phenomena, has been widely discussed elsewhere, but less so for foam polymers or in the context of imaging-based radiation exposure. This work describes, for the first time, the dosage response of a commercially-available impact protection foam in the context of structure-properties characterization with micro-computed X-ray tomography. These observations are informative across design and engineering, mechanics and material science domains. Engineers using heuristic materials selection techniques [16] for impact protection should consider changes in properties due to radiation sensitivity for space, nuclear or other radiation-prone applications. Mechanicians and material scientists are cautioned to take care when conducting structure-property imaging of polymer materials, particularly foamed or hierarchical form, with unknown dose sensitivity. In an exemplar case of the measurement altering the measurand unexpectedly, even relatively modest doses from X-ray CT can effect the polymer enough to significantly change thermomechanical properties (T_*g*_, E′, E″). Experiments showed as much as a 4× increase in room temperature stiffness and 2× increase in dissipation. Despite this, chemical structure changes (oxidization, crosslinking) remained below detection limits of routine characterization techniques. This serves as an initial step for polymer scientists to investigate more thoroughly the nature of the reaction process leading to changes in properties and to develop improved chemistry to modify the dose-response in vinyl nitrile blends. Designers and product engineers may seek to use radiation processing as a straight-forward means to adjust thermomechanical properties, for example to optimize energy dissipation in functionally graded materials [71]. And to this end, the mechanical model presented here captures the time-dependant properties of the foam as a function of temperature and dose, and the open-source user material is a tool for finite element-based computational design.

## Supplementary Material

SI

## Figures and Tables

**Fig. A.8.**
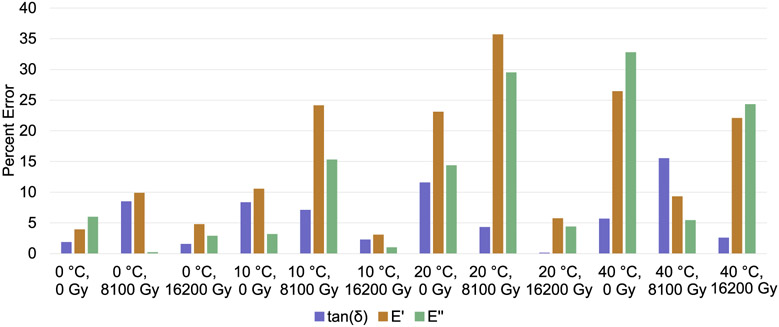
Percent error between simulated and experiment DMA values for a range of temperatures and doses. Although imperfect, the model captures the general trends in behavior.

## Data Availability

The raw data required to reproduce these findings are available to download from https://doi.org/10.18434/mds2-2989. The processed data findings, including basic analysis scripts for experiments, figure generation, and the user material implementation, are also available in that repository. Additionally, the user material implementation is available on the NIST GitHub: https://github.com/usnistgov/viscoelastic_SLS_umat_with_radiation_stiffening.

## Footnotes

1 Certain commercial equipment, instruments, or materials are identified in this paper in order to specify the experimental procedure adequately. Such identification is not intended to imply recommendation or endorsement by NIST, nor is it intended to imply that the materials or equipment identified are necessarily the best available for the purpose.

## Appendix A. Viscoelastic model: theory, calibration, and validation

A small-strain linear viscoelastic finite element user material model that captures the temperature and dose effects of the vinyl nitrile foam is described. The derivation of the finite element implementation and the fitting routine are detailed here. The model assumes small strain linear viscoelasticity as described by a standard linear solid (SLS). The standard linear solid model is equivalent to the generalized Maxwell with N = 1 Maxwell elements. Additional assumptions, based on observation of the foam behavior, include: 1) no thermo-mechanical coupling or implicit thermal or dose changes during an experiment because internal heating is negligible and any change in temperature or dose must be explicitly defined element-by-element, 2) rate independent elastic modulus varies linearly with dose, and 3) the Poisson’s ratio is a constant value and fixed with respect to temperature and dose, in addition to the typical assumptions made in implicit finite element modeling.

### A.1. Theory

The 1D ordinary differential equation of the SLS model is given by,

(A.1)
$$\sigma +\frac{\eta_{1}}{E_{1}}\dot{\sigma }=E_{0}\epsilon +\frac{\eta_{1}\left(E_{0}+E_{1}\right)}{E_{1}}\dot{\epsilon }$$

where σ is the total stress, ϵ is the total strain, $\eta_{1}$ is the damping viscosity of the dashpot and $E_{1}$ is the stiffness of the spring in the Maxwell element, and $E_{0}$ is the stiffness of the main spring.^2^ A total viscoelasticity model is used for the 3D implementation, in contrast to a volumetric and isochoric split of the stresses or strain-energy invariant-based decomposition. Thus, the extension of Equation (A.1) to three-dimensions directly follows the implementation described by Kaliske and Rothert [69]. In the time-discretized framework, the stress tensor at the next time increment, t=n+1 can be broken into two components,

(A.2)
$$\sigma^{n+1}=h_{e}^{\;n+1}+h_{1}^{\;n+1}$$

where $h_{e}^{\;n+1}$ is the elastic stress of the main spring, and $h_{1}^{\;n+1}$ is the internal stress related to the spring-dashpot Maxwell element.

Next, the update equation for $h_{e}^{\;n+1}$ is

(A.3)
$$h_{e}^{\;n+1}=h_{e}^{\;n}+\Delta h_{e}=h_{e}^{\;n}+C_{e}\Delta \varepsilon$$

where Δε is the total increment in strain, and $C_{e}$ is the Hookean elastic stiffness tensor, which can be expressed using two material parameters: Poisson’s ratio (ν) and Young’s modulus ($E_{0}$). In this implementation, the Young’s modulus corresponds to the stiffness of the main spring as denoted in Equation (A.1). In Equation (A.3), it is assumed that the current increment in total strain, Δε, is provided as input to the stress-update subroutine.

The update equation for $h_{1}^{\;n+1}$ is,

(A.4)
$$h_{1}^{\;n+1}=\text{exp}\left(-\frac{\Delta t}{\tau_{1}}\right)h_{1}^{\;n}+\gamma_{1}\frac{1-\text{exp}\left(-\frac{\Delta t}{\tau_{1}}\right)}{\frac{\Delta t}{\tau_{1}}}\Delta h_{e}$$

where $\tau_{1}$ and $\gamma_{1}$ are material parameters, and Δt is the increment in time (i.e., $\Delta t=t^{n+1}-t^{n}$). The parameter $\tau_{1}$ is the SLS relaxation time and it is defined as,

(A.5)
$$\tau_{1}≔\frac{\eta_{1}}{E_{1}}$$

where $\eta_{1}$ is the viscosity of the damper. The $\gamma_{1}$ term is the stiffness of the Maxwell spring normalized by the main spring,

(A.6)
$$\gamma_{1}≔\frac{E_{1}}{E_{0}}$$

With the stress at the next time step, $\sigma^{n+1}$, now fully defined, the final step is to calculate the algorithmic tangent modulus, $C_{alg}$, which is necessary for implicit finite element schemes,

(A.7)
$$C_{alg}\equiv \frac{\partial \sigma^{n+1}}{\partial \varepsilon^{n+1}}=\left[1+\gamma_{1}\frac{1-\text{exp}\left(\frac{-\Delta t}{\tau_{1}}\right)}{\frac{\Delta t}{\tau_{1}}}\right]C_{e}$$

### A.2. Calibration

In total, there are four SLS material parameters that must be calibrated: ν, $E_{0}$, $\tau_{1}$, and $\gamma_{1}$. In this implementation, some of these material parameters vary as a function of temperature, T, and radiation dosage, D. Poisson’s ratio is primarily a function of material structure in the small-strain regime [19] and thus is assumed to be constant with respect to changing temperature and dosage. It was computed with data from the same neat material in [35] using the mean value from 0.1 % to 5.1 % axial engineering strain. The resultant measurement was approximately ν=0.23, comparable to the idealized value of ν=0.25 [19]. The stiffness of the main spring, $E_{0}$, was assumed to vary primarily in proportion to radiation dosage, i.e., $E_{0}(D)$ and was computed directly from the measured stress and strain during stress relaxation experiments (shown in Fig. 1d) at 1600 s for each dose level ($E_{0}={\sigma (t)∕\epsilon (t)|}_{t=1600\;s}$). The remaining parameters were calibrated across a range of temperatures and dosages, $\tau_{1}(T,D)$) and $\gamma_{1}(T,D)$. The fitted regime spans approximately 253 K to 333 K and 0.0 kGy to 16.2 kGy.

The mean of the storage and loss moduli measurements as a function of dose and temperature (see Fig. 4), were used to compute $\gamma_{1}$ and $\tau_{1}$ via $\gamma_{1}=E^{\prime }∕E_{0}(1+\omega^{2}\tau^{2})∕(\omega^{2}\tau^{2})$ and $\tau_{1}=1∕(\omega \tan\;\delta )$. The $E_{0}$ dose response was fit in a least-squares sense to a 1^*st*^-order polynomial (R^2^ = 0.9917). Due to the sensitivity of the model with respect to small variations in $\gamma_{1}$ and $\tau_{1}$, the temperature-dose response surfaces for $\gamma_{1}$ and $\tau_{1}$ were fit to *7*^*th*^-order polynomial surfaces with an additional hyperbolic tangent term to account for the asymptotic behavior as the polymer transitions into the rubbery regime. These fitting functions were directly implemented in the user material to compute estimated material properties within the fitted range. The model requires parameters of temperature and dose to be specified for each element it is applied to in the simulation, typically by assigning the properties to one or more element sets in the input file.

For completeness, the explicit definitions of the calibrated SLS material parameters are summarized below. The polynomial surface used to model $\gamma_{1}$ as a function of temperature and dosage takes the following form,

(A.8)
$$f_{8}(T,D)=a_{80}\;\tanh\;(a_{81}T+a_{82})+a_{83}\;\tanh\;(a_{84}D+a_{85})\;f_{7}(T,D)=a_{70}T^{7}+a_{61}T^{6}D\;f_{6}(T,D)=a_{60}T^{6}+a_{51}T^{5}D\;f_{5}(T,D)=a_{50}T^{5}+a_{41}T^{4}D\;f_{4}(T,D)=a_{40}T^{4}+a_{31}T^{3}D\;f_{3}(T,D)=a_{30}T^{3}+a_{21}T^{2}D\;f_{2}(T,D)=a_{20}T^{2}+a_{11}TD\;f_{1}(T,D)=a_{10}T+a_{01}D\;f_{0}(T,D)=a_{00}$$

where $a_{ij}$ coefficients to be calibrated in the fitting routine. The $\gamma_{1}(T,D)$ function given by the summation

(A.9)
$$\gamma_{1}(T,D)=f_{8}+f_{7}+f_{6}+f_{5}+f_{4}+f_{3}+f_{2}+f_{1}+f_{0}.$$

The polynomial surface used to model $\tau_{1}$ takes the following form,

(A.10)
$$g_{5}(T,D)=b_{50}T^{5}+b_{41}T^{4}D\;g_{4}(T,D)=b_{40}T^{4}+b_{31}T^{3}D\;g_{3}(T,D)=b_{30}T^{3}+b_{21}T^{2}D\;g_{2}(T,D)=b_{20}T^{2}+b_{11}TD\;g_{1}(T,D)=b_{10}T+b_{01}D\;g_{0}(T,D)=b_{00}$$

where $b_{ij}$ are coefficients to be calibrated, and the $\tau_{1}(T,D)$ function is given by

(A.11)
$$\tau_{1}(T,D)=g_{5}+g_{4}+g_{3}+g_{2}+g_{1}+g_{0}.$$

Finally, $E_{0}(D)$ was fitted in a least squares sense to a linear function of dosage via,

(A.12)
$$E_{0}(D)=c_{1}D+c_{0}$$

where $c_{i}$ are coefficients to be calibrated. All of the calibrated values for the coefficients in Equation (A.8), Equation (A.10), and Equation (A.12) are provided in Table A.1.

**Table A.1:** The calibrated coefficients for the SLS material parameters: $\gamma_{1}$, $\tau_{1}$, and $E_{0}$ (see Equation (A.9), Equation (A.11), and Equation (A.12)). These calibration coefficients are based in units of temperature (T) in Kelvin and dosage (D) in Grays. The calibrated range for temperature is 253 K ≤T≤ 333 K, and the calibrated range for dosage is 0 Gy ≤D≤ 16200 Gy. The fourth and final SLS material parameter was assumed to be constant, ν=0.23, and is therefore not shown in this table.

$\gamma_{1}(T,D)\;[\text{unitless}]$		$\tau_{1}(T,D)\;[s]$		$E_{0}(D)\;[\text{Pa}]$	
$a_{80}$	2.876 096 × 10^2^	$b_{50}$	−4.227 011 × 10^−9^	$c_{1}$	−7.311 166 × 10^−1^
$a_{81}$	1.000 000	$b_{41}$	7.794 590 × 10^−12^	$c_{0}$	2.425 156 × 10^4^
$a_{82}$	1.000 000	$b_{40}$	6.581 727 × 10^−6^		
$a_{83}$	−3.221 250 × 10^1^	$b_{31}$	−9.562 996 × 10^−9^		
$a_{84}$	1.000 000	$b_{30}$	−4.096 525 × 10^−3^		
$a_{85}$	2.178 455 × 10^1^	$b_{21}$	4.402 199 × 10^−6^		
$a_{70}$	−1.594 650 × 10^−13^	$b_{20}$	1.274 326		
$a_{61}$	3.907 026 × 10^−11^	$b_{11}$	−9.016 026 × 10^−4^		
$a_{60}$	2.570 538 × 10^−8^	$b_{10}$	−1.981 666 × 10^2^		
$a_{51}$	−6.972 097 × 10^−8^	$b_{01}$	6.935 014 × 10^−2^		
$a_{50}$	−3.411 580 × 10^−5^	$b_{00}$	1.232 661 × 10^4^		
$a_{41}$	5.170 611 × 10^−5^				
$a_{40}$	1.641 764 × 10^−2^				
$a_{31}$	−2.039 644 × 10^−2^				
$a_{30}$	−2.863 609				
$a_{21}$	4.513 288				
$a_{20}$	−1.908 709 × 10^2^				
$a_{11}$	−5.311 418 × 10^2^				
$a_{10}$	1.222 954 × 10^5^				
$a_{01}$	2.597 090 × 10^4^				
$a_{00}$	−1.097 881 × 10^7^				

### A.3. Validation

The above UMAT and polynomial model fit was validated against several experimental conditions, using a virtual DMA experiment, whereby cyclic displacement was applied to the top surface of a cylindrical specimen (modeled as a quarter cylinder with symmetry boundaries on the cut faces) while the bottom surface was held fixed. The response was computed at the center element of the model, and the differences between applied and calculated sinusoids used to calculate tan δ, E′ and E″. In this way, the computed viscoelastic parameters could be directly compared to experimental values, if temperature and dose parameters were chosen to correspond to experimental conditions. Fig. A.8 shows the absolute percent error (% *Error* = (∣*simulation* − *experiment*∣/*experiment*) 100%) for twelve different temperature-dose pairs.
