# Supplementary material for: Unintended consequences: Assessing thermo-mechanical changes in vinyl nitrile foam due to micro-computed X-ray tomographic imaging
Source: Mater Des. Author manuscript; Available in PMC 2023 Nov 30. (PMC10688219; doi:10.1016/j.matdes.2023.112381)
Supplement: SI [file NIHMS1938427-supplement-SI.pdf]

Supplemental Information for *Unintended consequences: Assessing thermo-mechanical changes in vinyl nitrile foam due to micro-computed X-ray tomographic imaging*

Alexander K Landauer, Zois Tsinas, Orion L Kafka, Newell H Moser, Jack L Glover, Aaron M Forster

Data:

The raw and processed data and analysis scripts used for the publication are available with documentation from:

https://datapub.nist.gov/od/id/mds2-2989

Code:

The finite element user material implementation is available on the NIST GitHub:

https://github.com/usnistgov/viscoelastic_SLS_umat_with_radiation_stiffening
